# *In Vitro* Identification and *In Vivo* Confirmation of Inhibitors for *Sweet Potato Chlorotic Stunt Virus* RNA Silencing Suppressor, a Viral RNase III

**DOI:** 10.1128/JVI.00107-21

**Published:** 2021-05-24

**Authors:** Linping Wang, Sylvain Poque, Karoliina Laamanen, Jani Saarela, Antti Poso, Tuomo Laitinen, Jari P. T. Valkonen

**Affiliations:** a Department of Agricultural Sciences, University of Helsinki, Helsinki, Finland; b Institute for Molecular Medicine Finland, University of Helsinki, Helsinki, Finland; c School of Pharmacy, University of Eastern Finland, Kuopio, Finland; d Department of Internal Medicine VIII, University Hospital Tübingen, Tübingen, Germany; University of Maryland, College Park

**Keywords:** viral RNase III, RNA silencing suppressor, inhibitor identification, high-throughput screening, FRET, synergism, sweetpotato, SPCSV

## Abstract

Sweet potato virus disease (SPVD), caused by synergistic infection of *Sweet potato chlorotic stunt virus* (SPCSV) and *Sweet potato feathery mottle virus* (SPFMV), is responsible for substantial yield losses all over the world. However, there are currently no approved treatments for this severe disease. The crucial role played by RNase III of SPCSV (CSR3) as an RNA silencing suppressor during the viruses’ synergistic interaction in sweetpotato makes it an ideal drug target for developing antiviral treatment. In this study, high-throughput screening (HTS) of small molecular libraries targeting CSR3 was initiated by a virtual screen using Glide docking, allowing the selection of 6,400 compounds out of 136,353. We subsequently developed and carried out kinetic-based HTS using fluorescence resonance energy transfer technology, which isolated 112 compounds. These compounds were validated with dose-response assays including kinetic-based HTS and binding affinity assays using surface plasmon resonance and microscale thermophoresis. Finally, the interference of the selected compounds with viral accumulation was verified *in planta*. In summary, we identified five compounds belonging to two structural classes that inhibited CSR3 activity and reduced viral accumulation in plants. These results provide the foundation for developing antiviral agents targeting CSR3 to provide new strategies for controlling sweetpotato virus diseases.

**IMPORTANCE** We report here a high-throughput inhibitor identification method that targets a severe sweetpotato virus disease caused by coinfection with two viruses (SPCSV and SPFMV). The disease is responsible for up to 90% yield losses. Specifically, we targeted the RNase III enzyme encoded by SPCSV, which plays an important role in suppressing the RNA silencing defense system of sweetpotato plants. Based on virtual screening, laboratory assays, and confirmation *in planta*, we identified five compounds that could be used to develop antiviral drugs to combat the most severe sweetpotato virus disease.

## INTRODUCTION

Sweetpotato is one of the most important staple crops worldwide. More than 30 viruses from 9 families have been reported to infect this crop (1). The most devastating and widespread sweetpotato disease, referred to as sweet potato virus disease (SPVD), is caused by synergism between two viruses, *Sweet potato chlorotic stunt virus* (SPCSV) (genus *Crinivirus*) and *Sweet potato feathery mottle virus* (SPFMV) (genus *Potyvirus*). This disease leads to yield reductions of up to 90% (2, 3). Moreover, SPCSV can establish synergistic effects with viruses from different genera (e.g., genera *Ipomovirus*, *Cucumovirus*, and *Carlavirus*), causing aggravated symptoms compared to their respective single infections (4). Conventional plant virus control strategies, such as virus resistance breeding and regeneration of virus-free tissue, have been attempted to control these synergistic diseases; however, no durable resistant cultivars have been developed or identified thus far. Furthermore, the combination of virus-free material with prophylactic measures only slightly limited the spread of SPCSV-mediated synergistic diseases (4–7). Therefore, in this study, we explored a new strategy to control SPVD, and possibly other SPCSV-mediated synergistic diseases, by identifying inhibitors for the key viral protein of SPCSV that causes synergistic infection.

RNA silencing induced by double-stranded RNA (dsRNA) is a natural antiviral defense response commonly found in both plants and animals (8–10). However, some viruses encode one or more proteins that act as RNA silencing suppressors to counteract the host’s antiviral defense by interacting with components involved in RNA silencing (11, 12). The mechanism behind the synergism between SPCSV and other viruses was shown to be linked to a highly conserved RNase III encoded by SPCSV (CSR3), which suppresses RNA silencing by cleaving small interfering RNAs (siRNAs) and therefore preventing the host antiviral RNA silencing response (13–15). The genome of SPCSV is composed of two single-stranded plus-sense RNA segments (RNA1 and RNA2) harboring five and seven open reading frames (ORFs), respectively (16); CSR3 is encoded by the third ORF from the 3′ end of the RNA1 segment. Since CSR3 was shown to be critical for the establishment of SPCSV viral synergism and disease development, CSR3 was seen as a promising target for antiviral drug discovery.

RNase III enzymes are divided into three classes according to the presence of certain domains (17). Functional and structural studies have shown that CSR3 belongs to class 1 RNase III enzymes, which contain a single endonuclease domain (endoND) and a dsRNA-binding domain (dsRBD). Members of this class are found in bacteria, fungi, and viruses (13, 18) and have been well characterized in Escherichia coli, Aquifex aeolicus, Thermotoga maritima, and Mycobacterium tuberculosis, for which several high-resolution crystal structures are available in the Protein Data Bank (PDB) (19, 20). Considering the important role of CSR3 (an RNA silencing suppressor) in SPCSV-mediated synergistic interactions and the availability of structural information on class 1 RNase III, the identification of inhibitors could be seen as a promising approach to control SPVD. However, to date, no compounds have been reported to exhibit any antagonism against CSR3 activity. In this study, we performed the first systematic CSR3 inhibitor screening and identification using both *in vitro* and *in vivo* methods. These findings will serve as a promising starting point for the development of effective treatments for virus diseases in sweetpotato plants.

## RESULTS

### High-throughput screening strategy.

**FIG 1:**
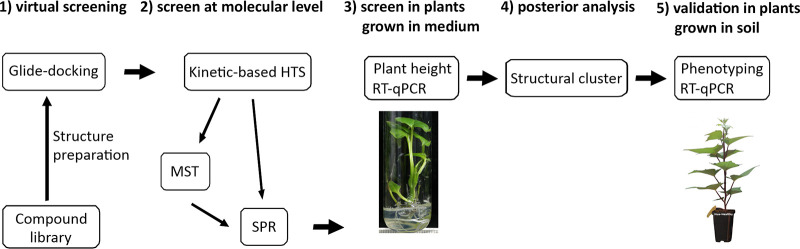
Workflow of CSR3 inhibitor identification.

The identification of CSR3 inhibitors was carried out in five phases (Fig. 1). In phase 1, the structure of CSR3 was modeled, and virtual screening using Glide docking was performed with 136,353 compounds that target the active site of CSR3. In phase 2, compound screening in the laboratory was first performed with a kinetic-based high-throughput screening (HTS) method that we developed using fluorescence resonance energy transfer (FRET) technology. Next, the binding affinity between the compounds and CSR3 was characterized using two complementary assays, microscale thermophoresis (MST) and surface plasmon resonance (SPR). Phase 3 involved an *in vitro* screening assay using coinfected (SPCSV and SPFMV) sweetpotato plants grown in culture medium. The inhibitors’ impact on plant growth was measured using plant height, and their effects on viral accumulation were monitored by measuring the relative abundances of the transcripts encoding viral coat proteins using reverse transcription-quantitative PCR (RT-qPCR). Phase 4 consisted of a posterior cluster study of the compounds based on the compound structures. Finally, phase 5 involved validation assays *in planta* of plants grown in soil using a plant phenotyping platform and the relative expression of viral coat proteins.

### Structural modeling and virtual screening.

**FIG 2:**
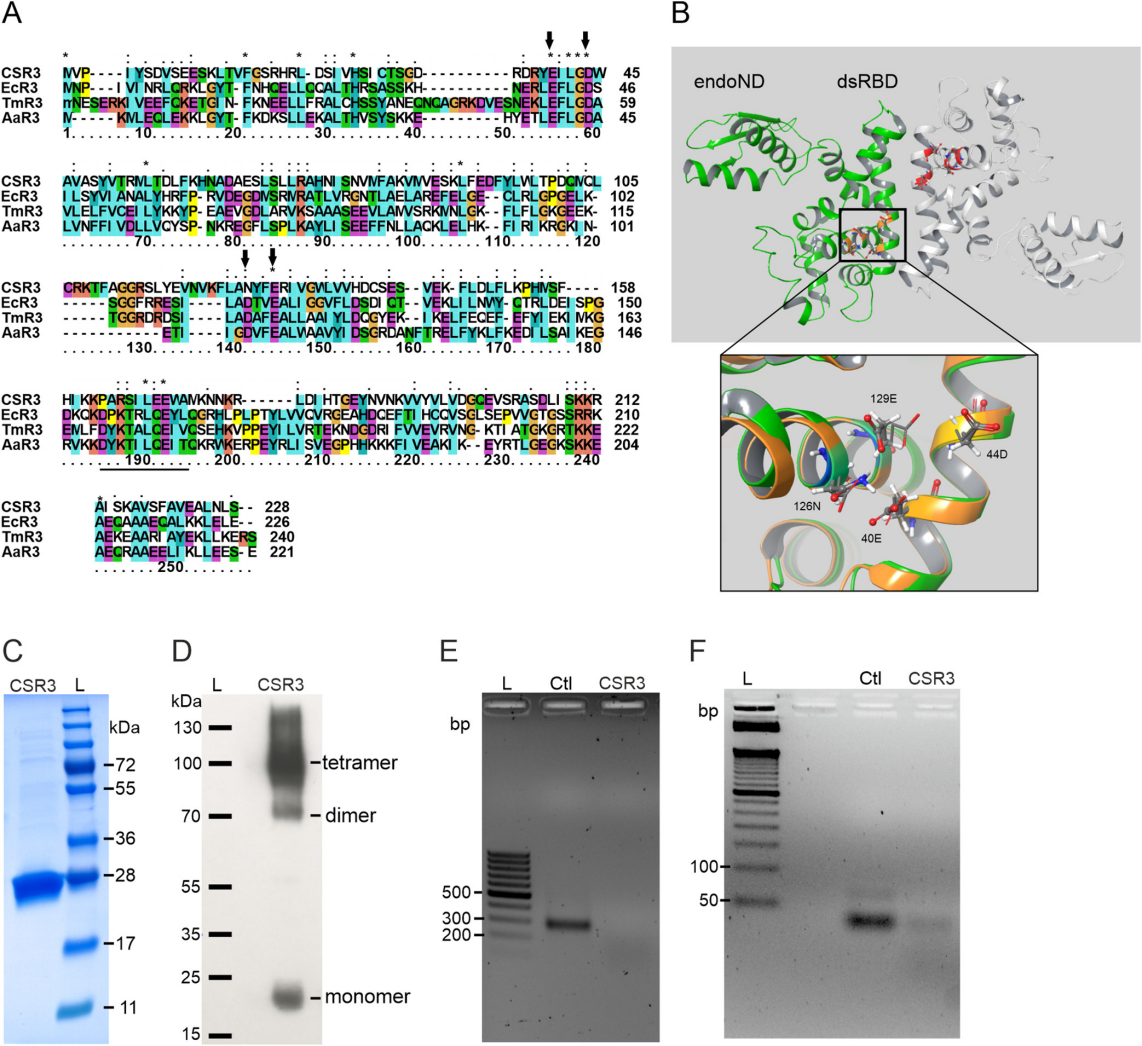
Homology modeling, purification, and characterization of CSR3. (A) Amino acid sequence alignment of CSR3 and EcR3, TmR3, and AaR3 was performed using MAFFT. The asterisks, colons, and periods indicate fully conserved, strongly similar, and weakly similar residues, respectively, between groups. Their active sites are indicated with black arrows. (B) The modeled structure of CSR3, a dimer, was constructed using I-TASSER. The two monomers of CSR3 are represented in green and gray. Each CSR3 monomer is composed of an endoND and a dsRBD. The superimposed structures of the endoND active sites of CSR3 and AaR3 (PDB accession number 2NUG) are highlighted in the higher-magnification view. The active site of CSR3 contains 4 amino acid residues (40E, 44D, 126N, and 129E), which are represented by tubes, and the corresponding amino acid residues of AaR3 are represented by ball-and-stick models. (C) CSR3 (∼26 kDa) purified for HTS was analyzed by SDS-PAGE. (D) Native Western blotting using rabbit polyclonal antibodies against CSR3. (E and F) CSR3 activity was assessed by agarose gel electrophoresis following incubation of 200-bp (E) and 22-bp (F) dsRNAs at 37°C for 40 min either with CSR3 or without any endoribonuclease enzyme (control [Ctl]). DNA or protein ladders are indicated by L.

The amino acid sequence analysis revealed that CSR3 (228 residues) is rather similar in size to RNase III from E. coli (EcR3), A. aeolicus (AaR3), and T. maritima (TmR3), comprised of 226, 221, and 240 residues, respectively (Fig. 2A). These three proteins are all prototypical class 1 RNase III enzymes that have been well studied both structurally and functionally (13, 19, 20). I-TASSER identified the template’s structure by using the LOMETS server and then selected and scored the templates with the highest significance in the threading alignments, which were used to simulate a pool of protein structure decoys. Finally, the top five models were identified according to pairwise structure similarity using the program SPICKER (21). In our study, the top identified template structures consisted of the PDB structures under accession numbers 1O0W, 5B16, 3C4T, 2EB1, 2A11, 2FFI, 3O2R, 2NUG, 1YYK, and 4CE4. We selected the highest-ranked CSR3 model (I-TASSER c score, 0.56; TM score, 0.79 ± 0.09), which is composed of an endoND and a dsRBD connected by a flexible linker that is similar to those of other class 1 RNase III enzymes (Fig. 2B).

The catalytic activity of RNase III is mediated by two metal ions for which the side chains of the 4 amino acid residues are negatively charged or can be deprotonated, allowing the attraction of positively charged metal ions (e.g., Mg^2+^), which further attract the negatively charged phosphate groups of dsRNA (22). The catalytic site of CSR3 is composed of the 4 amino acid residues 40E, 44D, 126N, and 129E (Fig. 2A), whereas 107D in AaR3 corresponds to 126N in CSR3; a superimposed image of the catalytic sites of CSR3 and AaR3 (PDB accession number 2NUG) (1.7 Å) is shown in Fig. 2B. These results were consistent with those of previous studies demonstrating that these 4 amino acid residues were essential for the catalytic activity of class 1 RNase III (20, 23). Moreover, previous studies showed that mutations of CSR3 40E and 44D lead to a loss of function of its endoribonuclease activity on dsRNA and suppression of RNA silencing (15, 18, 24), confirming their critical function for CSR3 activity.

In this study, the structures of 136,353 small molecules obtained from the High Throughput Biomedicine Unit of the Institute for Molecular Medicine Finland (HTB-FIMM) compound libraries were prepared with the LigPreg function of Schrödinger Maestro using the default setup conditions (25). Residues of the CSR3 activity site were selected as the center of the Glide-Grid box, and docking was performed using SP and XP scoring modes using the OPLS3 force field (26). Since the virtual screening was done using a structural model, we selected a relatively large number of compounds (6,400) according to their GlideScore rank order for further laboratory screening (their docking scores are listed in Table S1 in the supplemental material).

### Preparation and characterization of the enzyme.

His-tagged full-length CSR3 (GenBank accession number ADQ42569.1) was expressed in E. coli and purified with a nickel-nitrilotriacetic acid (Ni-NTA) affinity column. The size of recombinant CSR3 was ∼26 kDa, as shown in Fig. 2C. Under native Western blotting conditions, CSR3 could be detected as a monomer and a dimer (Fig. 2D); under denatured conditions, CSR3 was shown to be primarily monomeric in our previous study (24, 27), which is consistent with the results of SDS-PAGE in Fig. 2C. Although CSR3 could also exist in a tetrameric conformation in native Western blots (27), several studies have shown that the homodimer is the biologically active conformation (20, 28, 29). Theoretically, CSR3 could cleave any size of dsRNA *in vitro*; thus, its enzymatic activity was evaluated using either 22-bp siRNA or 200-bp dsRNA. Our results demonstrated that both substrates could be cleaved into smaller fragments by the purified CSR3 (Fig. 2E and F, respectively), demonstrating its activity.

### Kinetic-based HTS.

**FIG 3:**
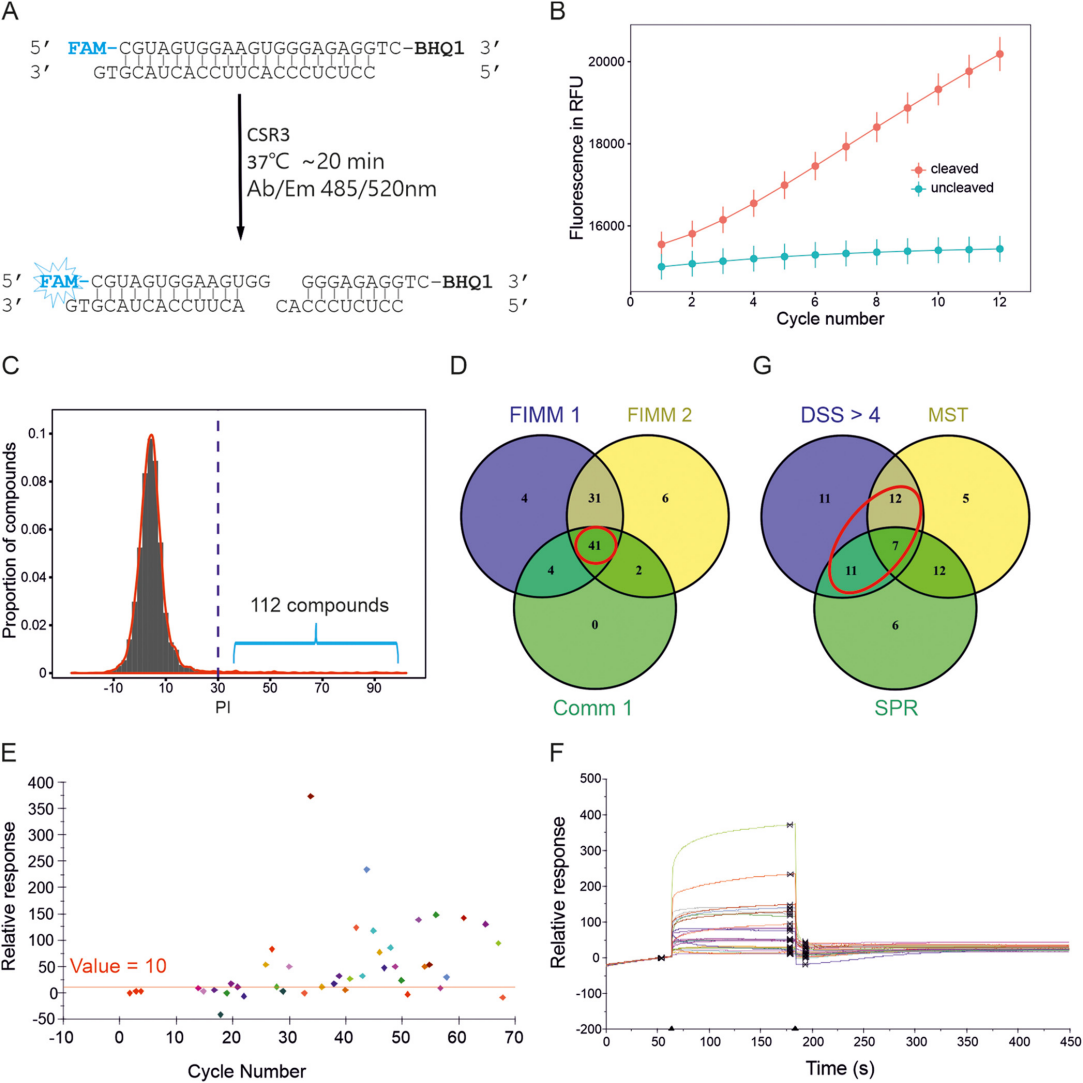
Inhibitor screening at the molecular level. (A) Schematic diagram of the kinetic-based HTS assay using FRET technology, which represents a 22-bp labeled siRNA cleaved by CSR3 to generate a fluorescence signal. (B) One-concentration screening using the kinetic-based HTS assay. Fluorescence values (in relative fluorescence units [RFU]) as a function of the detection cycle number (∼17 min total) at 37°C show the difference between the positive-control (uncleaved) and negative-control (cleaved) reactions; values are the means ± standard deviations (SD) (*n* = 240) from 20 screening plates. (C) PI distribution of the 6,622 compounds. (D) Dose-response results using the kinetic-based assay. The Venn diagram shows the 41 candidates selected from three independent assays using different compound sources: FIMM libraries (FIMM 1 and FIMM 2) or commercial compounds (Comm 1). (E and F) Results of binding stability of screening compounds using SPR, with a threshold of relative response (RU) value of >10 (E), and their corresponding SPR sensorgrams (F). (G) Results of the binding affinity assays using MST and SPR highlighting the 30 candidates selected from the kinetic-based assays.

Based on the catalytic activity of CSR3 on 22-bp siRNA, our kinetic-based HTS assay was designed using distance-dependent FRET technology. Specifically, one strand of siRNA, as a substrate, was labeled with a fluorescent dye (6-carboxyfluorescein [FAM]) at the 5′ end and a quencher (black hole quencher 1 [BHQ1]) at the 3′ end. The initial reaction rate of CSR3 was quantified using the slope of raw fluorescence under an excitation/emission spectrum at 485/520 nm (Fig. 3A). According to our previous study (24) detailing CSR3 kinetic-based HTS development and optimization, this assay was conducted using 100 nM CSR3 enzyme and 375 nM siRNA substrate over 12 plate read cycles (∼17 min total) at 37°C. In this study, a total of 6,622 compounds (including 6,400 selected from Glide docking and 222 empirically selected compounds for preliminary assay setup) were screened using kinetic-based HTS at a final concentration of 10 μM. All 384-well screening plates contained 32 negative (with CSR3 [i.e., the substrate was successfully cleaved]) and 32 positive (without CSR3 [i.e., the substrate remained intact]) control reaction mixtures. To evaluate the quality of the HTS assay, the Z-factor (Z′) parameter was calculated using the reaction rates of the positive and negative controls (30). The average Z′ was 0.82 ± 0.04 for the 20 screening plates (0.5 is generally an acceptable threshold for an excellent assay), indicating that the kinetic-based HTS was technically successful and that the results were qualitatively and quantitatively adequate. It also showed that the slopes of raw fluorescence between the negative and positive controls were clearly separated (Fig. 3B). Moreover, the effects of each compound on the reaction rate relative to both controls were used to calculate their percentage of inhibition (PI) (see the equation in “Data analysis,” below). PI values of the 6,622 compounds can be found in Table S2, from which a total of 112 compounds with a PI of >30% were selected for further dose-response testing (see the PI distribution of the 6,622 compounds in Fig. 3C).

### Dose-response assay.

A dose-response assay with six concentrations (1.25 nM to 50 μM) was carried out in three independent experiments, which included two tests with the 112 selected FIMM compounds (i.e., compounds prepared by the HTB-FIMM) and one test using the newly ordered commercial compounds (99 of the 112 compounds, i.e., compounds prepared by a second supplier). A dose-response curve was generated for each compound based on PI values as a function of the concentration. Along with the half-maximal inhibitory concentration (IC_50_), the drug sensitivity score (DSS) was determined for each compound and used for candidate selection (see dose-response results of the three repeats [FIMM 1, FIMM 2, and Comm 1] in Table S3). DSS is a parameter that integrates the five characteristics of dose-response curves (IC_50_, slope at IC_50_, minimum activity level, and top and bottom asymptotes) into a single metric to score the sensitivity of individual compounds, as described previously by B. Yadav et al. (31), which has been widely used for HTS assays. In this study, based on a DSS threshold of >4 (ranging from 0 to 22 in our study), 41 compounds were selected from the three independent assays (Fig. 3D, red circle).

### Binding affinity assays using MST and SPR.

Binding affinity assays were first carried out with the 99 commercial compounds using the MST method. As a result, 36 compounds of interest were identified based on three criteria: (i) a signal/noise ratio of >5, (ii) a response amplitude of >4, and (iii) a dissociation constant (*K_d_*) of <200 μM. The MST binding affinity results of the 36 compounds are listed in Table S4. Based on the results from the HTS and MST assays, 56 compounds were then screened using SPR at a concentration of 100 μM. Further dose-response testing over 12 concentrations (3 μM to 200 μM) was performed with 44 compounds that showed a relative response unit (RU) value of >10 (Fig. 3E and F). Based on the steady-state affinity (*K_D_*) and the kinetics in the dose-response assay, 36 compounds of interest were identified (binding affinity results of SPR can be found in Table S5). Altogether, at the molecular level, based on the results of the kinetic-based HTS assay and the union set of MST and SPR, 30 compounds were considered potential CSR3 inhibitors (Fig. 3G).

### Inhibitor screening in plants grown in culture medium.

Since tests in culture medium consume smaller amounts of compounds and can be carried out under relatively controlled conditions, sweetpotato plants coinfected with SPCSV and SPFMV were grown in medium supplemented with one of the 30 inhibitor candidates at a concentration of 50 μM. Their effects on plant growth were monitored by imaging the plants once a week. Two of the 30 compounds were excluded due to obvious stress symptoms such as deformation, wilting, bleaching, dried leaf margins, or severe growth defects, possibly because of their toxicity in plants. In addition, virus accumulation was quantified by measuring the relative abundance of the transcripts encoding viral coat proteins using RT-qPCR (32). The effect of individual compounds on virus accumulation was estimated by comparing treated and control plants.

**FIG 4:**
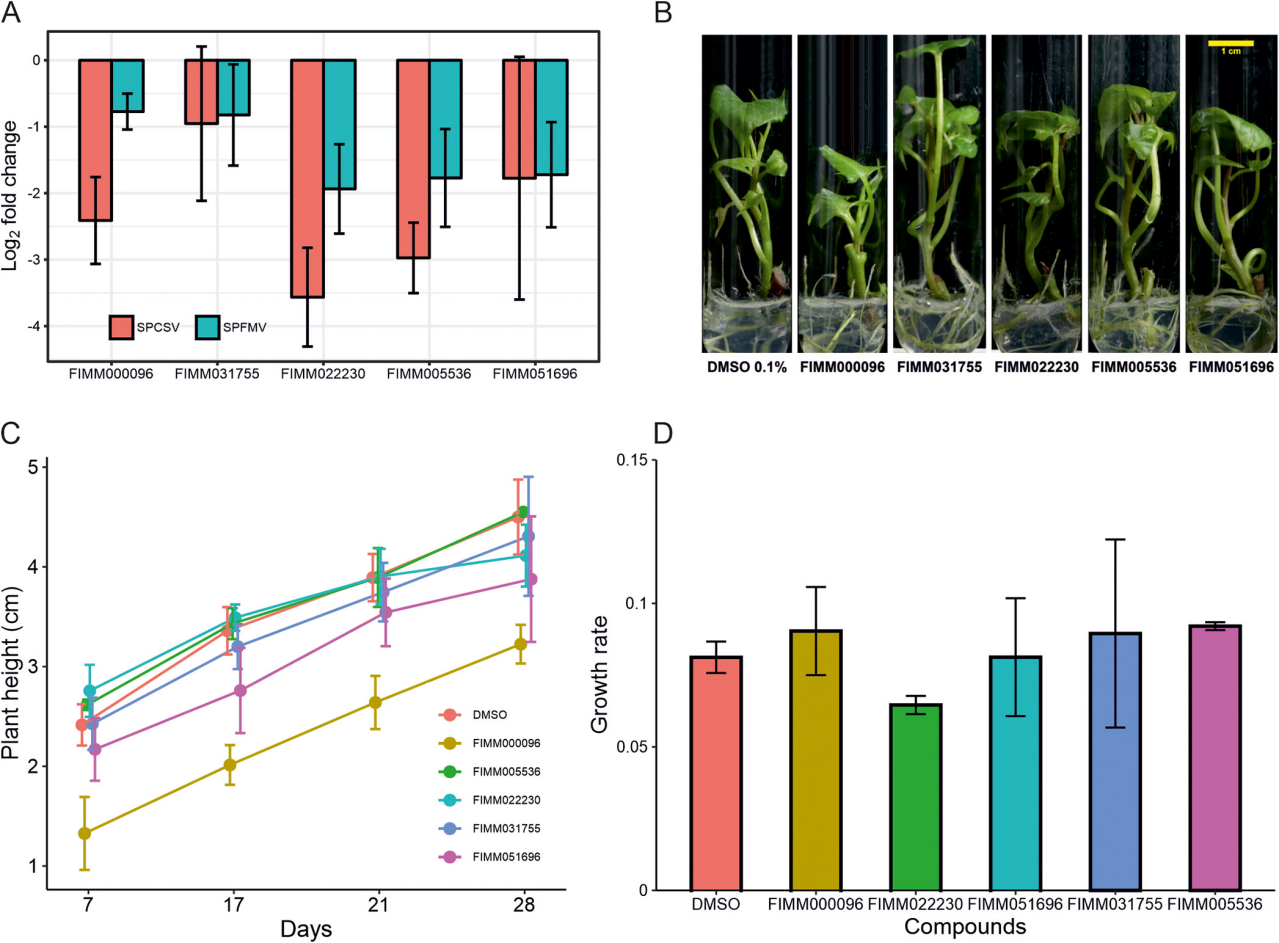
Results of inhibitor screening *in vivo*. (A) Reduction of SPCSV and SPFMV accumulation induced by the top five compounds using RT-qPCR, represented as the log_2_ fold change in the accumulation of the coat protein-encoding viral RNAs. Values are the means ± standard errors (SE) (*n* = 3 plants). (B) Representative pictures of plants grown in culture medium supplemented with the five compounds or with 0.1% DMSO for 28 days. (C) Effects of the five compounds on plant height were measured every week. Data are shown as the means ± SE (*n* = 3). (D) Growth rate calculated with plant height between days 7 and 28 as a function of days. There is no significant difference between treated plants and DMSO plants (control) according to a Dunnett test (confidence level of 0.95).

With a fold change of viral accumulation of <0.6, 7 and 11 compounds had negative effects on SPFMV and SPCSV accumulation in plants, respectively. Five common compounds showing negative effects on both SPFMV and SPCSV accumulation were selected (Table S6). Specifically, SPCSV accumulation was reduced approximately 8-fold by two compounds (FIMM022230 and FIMM005536), 4-fold by two compounds (FIMM051696 and FIMM000096), and 2-fold by the compound FIMM031755. SPFMV accumulation was reduced almost 4-fold by three compounds (FIMM022230, FIMM005536, and FIMM051696) and 2-fold by two compounds (FIMM000096 and FIMM031755) (Fig. 4A). Overall, these five compounds reduced both SPCSV and SPFMV accumulation without any phytotoxicity effects on sweetpotato plants (see experimental plant pictures in Fig. 4B and their effects on plant growth indicated by plant height over time in Fig. 4C and D).

### Structural clustering of the compounds.

**FIG 5:**
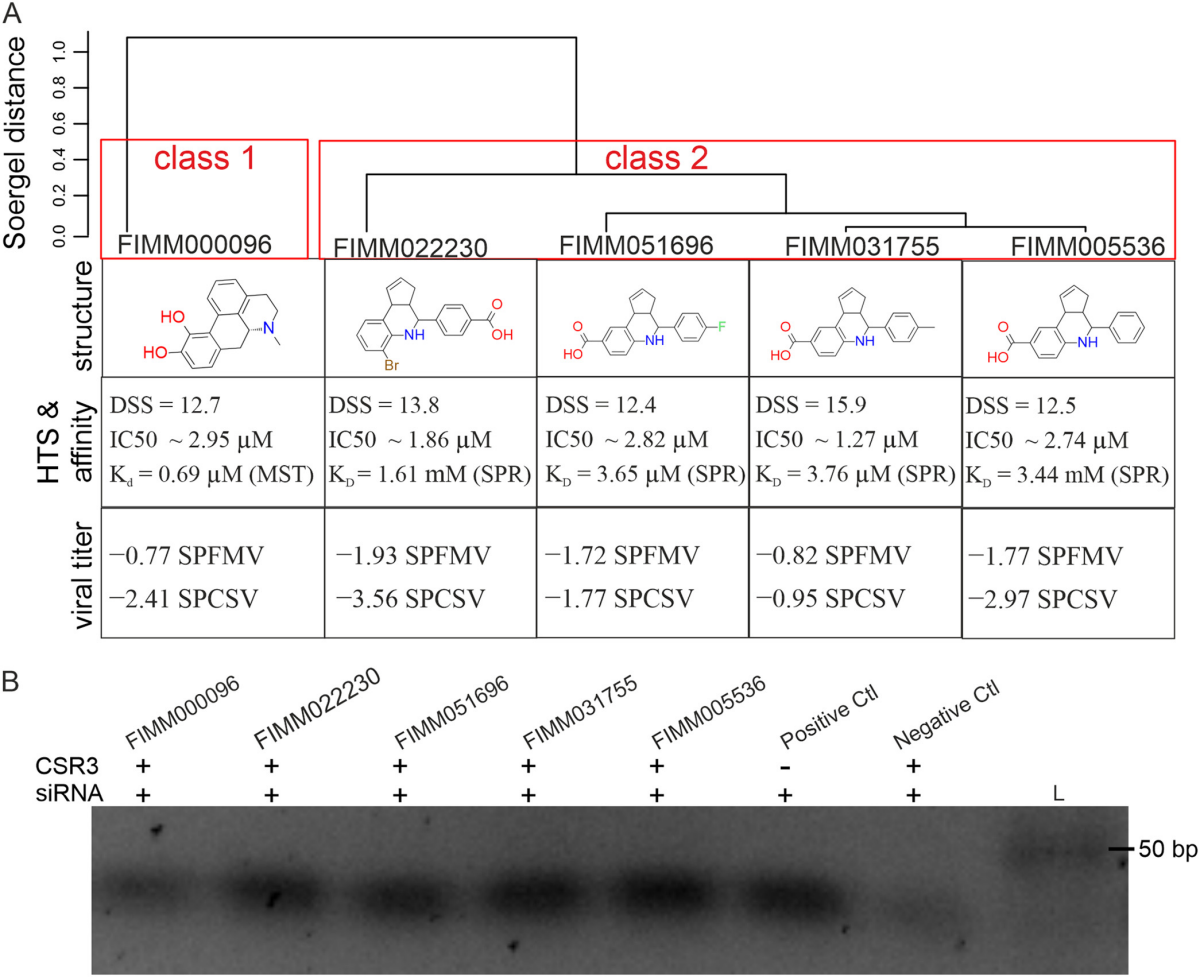
Hierarchical clustering of the selected compounds based on structure. (A) The five compounds were clustered into two classes using the Tanimoto coefficient, WardLinkage, and a threshold of 0.5 (ChemBioServer). The structure of the compounds and their IC_50_ and DSS values from kinetic-based HTS, their *K_d_* or *K_D_* values from affinity binding assays (by either MST or SPR), and their effects on viral accumulation (log_2_ fold change relative to controls) in plants are summarized. (B) Electrophoresis in a 2.5% agarose gel of labeled siRNA incubated for 30 min at 37°C with CSR3 and/or the five compounds. The composition of the reaction mixture is the same as that for HTS screening.

The five inhibitors were clustered hierarchically into two classes. FIMM000096 was placed into class 1, whereas the other four compounds (FIMM005536, FIMM031755, FIMM051696, and FIMM022230) were placed into class 2 and had highly similar structures (Fig. 5A; information on their molecular formulas, suppliers, molecular weights, and simplified molecular-input line-entry system (SMILES), etc., can be found in Table S7). Altogether, the kinetic-based HTS revealed that the compounds had similar DSS and IC_50_ values ranging from 12.4 to 15.9 and 1.27 to 2.95 μM, respectively. The reduction of virus accumulation in plants in the presence of the compounds resulted in a log_2_ fold change of −0.77 to −3.56 relative to the controls; the *K_d_* of MST or the *K_D_* of SPR values from the binding affinity experiments ranged from 0.69 μM to 3.44 mM for these compounds (Fig. 5A). Furthermore, as illustrated by agarose gel electrophoresis, labeled siRNAs remained intact in the reaction mixtures containing siRNA, CSR3, and any of the five compounds, which is similar to the positive control (containing siRNA alone), while siRNAs were fragmented and no clear bands could be observed in the negative control (siRNA incubated with only CSR3) (Fig. 5B). Overall, these results showed that all five compounds can prevent CSR3-mediated siRNA cleaving, validating their ability to inhibit the endoribonuclease activity of CSR3 in biochemistry.

### Inhibitor validation in plants grown in soil.

**FIG 6:**
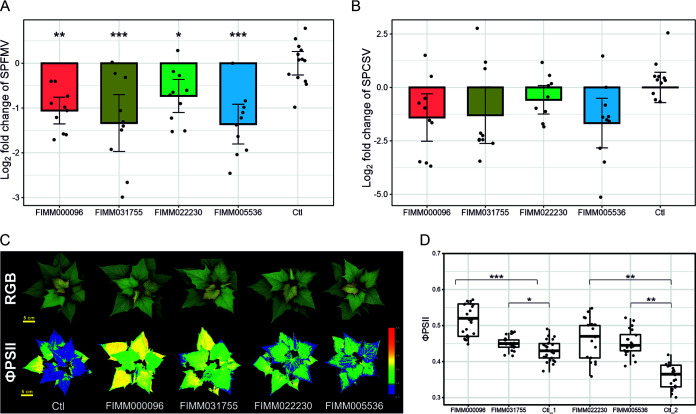
Inhibitor validation *in planta* using coinfected sweetpotato plants grown in soil. (A and B) The effects of four of the five compounds on viral accumulation were assessed in the coinfected sweetpotato plant by measuring the log_2_ fold change in coat protein expression of SPFMV (A) and SPCSV (B) compared to the water-treated plant (Ctl) using RT-qPCR. Data represent the means ± 95% confidence intervals (*n* = 10 plants). *, *P* < 0.05; **, *P* < 0.01; ***, *P* < 0.001 (based on Dunnett’s test for comparing several treatments with a control). (C) Top-view images of coinfected sweetpotato plants treated with one of the four compounds or water (Ctl) for 1 month. Photographs were obtained on day 41 by RGB imaging or ChlF imaging (ΦPSII). False-color images displaying ΦPSII values pixel by pixel were generated using a heat map color scale from dark blue to red, ranging from 0.3 to 0.8. (D) Overall quantum yield of PSII (ΦPSII) values of plants treated twice a week with one of the four compounds or water (Ctl_1 and Ctl_2) for 1 month. Experiments were carried out in two batches, each of which included a control condition and five plants per treatment. Values for ΦPSII were measured every day from day 37 to day 41 after plantlets were transferred to soil; these are shown in the box plots. *, *P* < 0.05; **, *P* < 0.01; ***, *P* < 0.001 (based on Dunnett’s test for comparing several treatments with a control).

Four of the five inhibitors (FIMM000096, FIMM031755, FIMM022230, and FIMM005536) were available in sufficient amounts to further confirm their effects on coinfected sweetpotato plants grown in soil using a plant phenotyping platform. Compounds were administered by regular foliar spray over 1 month. At the end of these treatments, we observed reductions of SPFMV accumulation (∼1.5- to 2-fold on average) and SPCSV accumulation (∼0.8- to 5-fold on average) in all four treatments compared to the untreated plants, although these reductions of viral titers, represented by log_2_ fold changes in coat protein expression, were statistically significant for SPFMV but not for SPCSV due to higher variability. Specifically, the log_2_ fold changes are −1.06, −1.33, −0.73, and −1.36 for SPFMV and −1.41, −1.30, −0.58, and −1.67 for SPCSV (Fig. 6A and B). This phenomenon is consistent with our previous conclusion that the more severe disease in coinfected plants is linked to an increase of the SPFMV titer (instead of the SPCSV titer), which is induced by CSR3 of SPCSV (33). At the morphological level, none of the treated plants showed signs of plant stress, as illustrated by the imaging results of the red, green, and blue (RGB) color mode in Fig. 6C.

At the physiological level, the quantum yield of photosystem II (ΦPSII) was used to monitor their effect on photosynthetic performance. Our previous data showed that ΦPSII, which indicates the proportion of light used by chlorophyll associated with PSII, is an efficient estimator of viral effects on sweetpotato plants (33). In this study, all four compounds caused a significant increase in ΦPSII values compared with the control values, reflecting an improvement of the photosynthetic performance in treated plants compared to untreated ones (Fig. 6D). Taken together, these results demonstrated that the four compounds had a positive effect on the photosynthetic performance of coinfected sweetpotato plants. Finally, a summary of all assay steps is shown in Table 1. Considering all screening steps, four compounds (hit rate of 0.0029%) were identified as inhibitors of CSR3.

**TABLE 1:** Summary of screening and validation of compounds

Step	No. of compounds tested	Screening concn	No. of compounds identified	Hit rate (%)
*In silico*	136,353	6,400	4.69
FRET	6,622	10 μM	112	1.69
FRET	99–112	1.25 nM–50 μM	41	37.6
MST	99	0.2 μM–400 μM	36	36.4
SPR	56	100 μM	42	75.0
SPR	42	3 μM–200 μM	36	85.7
*In vitro* screening (grown in medium)	30	50 μM	5	16.7
*In vitro* validation (grown in soil)	4	10 μM	4	100

Total	136,353	4	0.0029

## DISCUSSION

Currently, antiviral strategies in plants are based on either breeding virus-resistant cultivars or targeting viruses to prevent viral replication and spread (6, 34, 35). Typically, most virus control strategies are applied to preinfected plants, emphasizing the need to develop alternative antiviral strategies that are effective in postinfected plants. Indeed, searching for antiviral compounds that are capable of inhibiting essential steps in the virus life cycle may constitute a new means for counteracting disease development. Chemotherapy strategies widely used to treat animal viruses are rarely reported in plant virus studies (36). Nevertheless, RNA silencing suppressors, encoded by many viruses, have been shown to be essential for the collapse of antiviral defense (37–39). Moreover, the possibility of interfering with their activity was seen as a promising strategy to control viral diseases. RNA silencing suppressors of *Tombusvirus* P19 (binding to siRNAs and therefore preventing their incorporation into RNA-induced silencing complex) have been targeted in inhibitors studies using an electrophoretic mobility shift assay, SPR, and/or fluorescence detection on Ni^2+^-NTA plates. These studies have led to the identification of chemical inhibitors interfering with its binding activity (40–42).

Great interest in RNA interference (RNAi) research has been shown over the last 2 decades, and RNAi-related technologies remain crucial for developing crop protections against viruses (43). The engineering of virus-resistant crops has mainly focused on the integration of dsRNA coding for key viral proteins to trigger an RNAi defense response against targeted viruses. Moreover, recent studies showed that the exogenous application of RNA molecules (dsRNAs, siRNAs, hairpinRNAs [hpRNas], and microRNAs [miRNAs]) in plants through either coinoculation, agroinfiltration, or spraying can be sufficient to induce RNAi-mediated defense and eliminate virus accumulation up to 20 days posttreatment (44–46). Exogenous application of RNA molecules has been studied in many different viruses, such as *Pepper mild mottle virus* (PMMoV) (47), *Tobacco mosaic virus* (TMV) (48), and *Bean common mosaic virus* (BCMV) (45).

The present study focused on the identification of inhibitors of the RNA silencing suppressor CSR3 expressed by SPCSV, which, together with SPFMV, plays a central role in the development of the devastating viral disease in sweetpotatoes. CSR3 is an endoribonuclease belonging to the class 1 RNase III family. Until now, other endoribonucleases that have been targeted in inhibitor/activator identification include RNase H of HIV and a broad-spectrum antiviral RNase L of mammalian cells (49, 50). Although some RNase H inhibitors have been found, RNase H enzymes are functionally very different from class 1 RNase IIIs. For example, RNase H enzymes hydrolyze the RNA strands of DNA/RNA duplexes during reverse transcription (50). As expected, none of the RNase H inhibitors bind to CSR3 *in silico* (data not shown); thus, we employed a combination of *in silico* screening, kinetic- and affinity-based laboratory screening, and *in vivo* confirmation assays to identify inhibitors of the viral CSR3. As a result, five novel inhibitors of viral RNase III were identified, all of which showed the ability to negatively impact viral accumulation in sweetpotato.

Computer-aided molecular docking has played an important role in the early stages of drug discovery, allowing systematic calculation of ligand-protein interactions. Glide docking, used in our study, is a complete and hybrid method for searching for potential docking poses with high accuracy (51). Targeting the highly conserved active site of CSR3, as done in this study, could reduce the likelihood that resistance will develop within the virus population (52), which is important in the development of sustainable antiviral strategies. However, because a modeled CSR3 structure was used instead of a crystal structure, we screened a relatively large number (6,622) of small molecules with the kinetic-based assay.

Laboratory HTS in this study was performed using FRET technology. FRET-based methods have advantages, such as sensitivity and efficiency, but also disadvantages, as they are likely to produce false-positive and false-negative results (53, 54). In our study, false-negative findings were possible if compounds quenched the reporter fluorophore. False-positive results were possible under two conditions: (i) if compounds directly interacted with the substrate instead of CSR3 to prevent cleavage of the labeled siRNA and (ii) if compounds exhibited intrinsic fluorescence with absorption and emission spectra similar to those of the fluorophore reporter. To exclude false-positive results, two complementary methods, MST and SPR, were applied to directly measure the binding affinity between CSR3 and the studied compounds. MST is a fluorescence-based method used to record the motion of molecules in microscopic temperature gradients and detect changes in hydration shell, charge, or size (55); therefore, it is susceptible to disruption by intrinsically fluorescent compounds. However, SPR is used to monitor small changes in the optical reflective index at the sensor surface induced by an affinity interaction between the protein and the compound (56). If a compound does not properly dissociate from the sensor, it will affect the assay of the next analyte. Moreover, it is possible that certain small molecules will bind to the sensor surface (57, 58), resulting in a relatively wide range of *K_D_* values. For example, FIMM022230 had a high *K_D_* in the millimolar range for steady-state affinity (Fig. 5A).

To exclude inhibitors that interfere with endogenous RNase III and impact plant growth, a combination of molecular methods and imaging-based techniques was performed directly on sweetpotato plants grown in medium and/or soil. Most of the compounds (28 of 30) were not toxic to plants, and the accumulation of both SPCSV and SPFMV in plants grown in medium was reduced by five inhibitors. Structurally, these five compounds were clustered into two classes. These compounds were then used to further characterize existing molecules to identify optimal candidates by studying structure-activity relationships (59). Among the five inhibitors, the class 1 compound FIMM000096 has been approved as a powerful emetic and has also been used in the treatment of parkinsonism but with adverse effects (https://www.drugbank.ca/drugs/DB00714). To the best of our knowledge, the other four compounds, which belong to class 2, have not been reported either in the DrugBank database or for the treatment of viral diseases. However, they have been included in inhibitor screens for human enzymes or bacterial proteins according to their PubChem identifiers: compound identifier (CID) 2948389 (FIMM022230), CID 7114450 (FIMM031755), CID 2857906 (FIMM005536), and CID 4240943 (FIMM051696). They all were inactive in the studies except for FIMM031755, which affected the activity of chain B of the human cytokine/receptor binary complex (https://pubchem.ncbi.nlm.nih.gov/compound/7114450#section=Biological-Test-Results). Moreover, FIMM031755 has also been screened in our latest article about the development of a FRET-based screening method (24). The class 2 compounds, which share the same core structure but have different R groups, had different binding affinities, providing evidence for further analyses of structure-activity relationships. Although these compounds had beneficial effects on SPCSV- and SPFMV-infected sweetpotato plants grown in medium and soil, many other bioactivity, toxicity, and *in vivo* tests are still needed to develop antiviral agents that can be used in the field. The results reported here will aid in developing new strategies to combat the most severe and widespread sweetpotato viral disease, as four of the five candidates were also confirmed to positively impact plant performance by using a chlorophyll fluorescence (ChlF) imaging-based platform. Moreover, another similar RNase III has been found in *Pike-perch iridovirus* (PPIV) in fish (15); therefore, it is possible that more RNase III-like RNA silencing suppressors could be identified. Hence, our HTS method could be easily adapted for inhibitor identification for other viruses.

Until now, due to the difficulty in achieving broad viral resistance to different virus strains or genera, research studies have been focusing on approaches that could sustain protection against specific viruses, mainly using transgenic expression or exogenous application of RNA molecules to trigger RNAi-mediated plant defense mechanisms. Compared to those approaches that artificially induce RNAi defense to eliminate virus accumulation at the RNA level, the compounds (viricides) identified in this study directly target and inactivate a viral protein playing a key role in viral counterdefense. As all methods have their own merits and demerits, the exogenous application of RNA molecules is facing stability and suitability problems, while virus-resistant breeding, achieved by the generation of genetically modified organisms (GMOs), has raised considerable public concerns. Like pesticides, the application of viricides can be considered an attractive method considering its potential in those aspects. Besides, those compounds were selected for targeting a specific, highly conserved activity site, which could limit resistance-breaking events and allow further development for lower dosages, therefore reducing off-target effects. However, compound methods (viricides or pesticides) are associated with health risks for farmers, consumers, and the environment, which require extensive study before field application worldwide. Last but not least, for both exogenous methods (RNA molecules and compounds), costs for development and application will be a key factor toward practical application in the field.

## MATERIALS AND METHODS

### Protein expression, purification, and activity assay.

CSR3 (GenBank accession number ADQ42569.1) was fused to 6×His at its C terminus in the pET11d vector and expressed in E. coli BL21 (15, 18). Bacterial cells were cultured under selection with ampicillin (100 μg/ml) and chloramphenicol (25 μg/ml) at 37°C for 2 h (optical density [OD] of 0.5 to 0.6). CSR3 expression was induced with 0.1 mM isopropyl-β-d-1-thiogalactopyranoside (IPTG), and cells were harvested after 4 h at 37°C. Bacterial cells were first purified with Ni-NTA agarose (Qiagen, Venlo, Netherlands) and then lysed with lysozyme at a final concentration of 1 mg/ml (Sigma-Aldrich, St. Louis, MO, USA). The lysis, wash, and elution steps were performed using a His buffer kit (GE Healthcare, Chicago, IL, USA). The purified protein was stored in a buffer (20 mM Tris-HCl [pH 8], 50 mM NaCl, 10 mM MgCl_2_, 5% glycerol) using a buffer exchange column (PD MidiTrap G-25; GE Healthcare). Proteins were visualized on SDS-PAGE gels and quantified with a NanoDrop apparatus (Thermo Fisher Scientific, Waltham, MA, USA). The activity of CSR3 was tested using a 200-bp dsRNA substrate in a 20-μl reaction buffer (20 mM Tris-HCl [pH 8], 50 mM NaCl, 10 mM MgCl_2_); after incubation for 40 min at 37°C, the sample was analyzed on a 1% agarose gel.

### Homology modeling and virtual screening.

EcR3, TmR3, and AaR3 from E. coli, T. maritima, and *A. aeolicus*, respectively, were used for sequence alignment with MAFFT (60). The structural model of CSR3 was generated with I-TASSER (61). I-TASSER was used to identify the template’s structure using the LOMETS server and then to select and score the templates with the highest significance in the threading alignments, which were used to simulate a pool of protein structure decoys. Finally, the top five models were identified according to pairwise structure similarity using the program SPICKER (21). In our study, the top identified template structures consisted of the PDB structures under accession numbers 1O0W, 5B16, 3C4T, 2EB1, 2A11, 2FFI, 3O2R, 2NUG, 1YYK, and 4CE4. The highest-ranked model was selected for Glide docking analysis, which was processed with the Protein Preparation Wizard of Schrödinger (release 2016-4; Schrödinger LLC, NY, USA). The structures of compounds were prepared with the LigPreg function of Schrödinger using the default setup conditions. Four residues at the active site of CSR3 were selected as the center of the Glide-Grid box, and docking was performed using SP and XP scoring modes using the OPLS3 force field (26, 51). The top 6,622 compounds were selected based on the GlideScore rankings.

### Laboratory screening using FRET.

The HTS assay was designed based on FRET using a 22-bp siRNA (forward sequence, CGUAGUGGAAGUGGGAGAGGTC; reverse sequence, CCUCUCCCACUUCCACUACGTG) with a 2-nucleotide (nt) 3′ overhang labeled with the fluorescent dye 6-carboxyfluorescein (FAM) and a black hole quencher (BHQ1) on the sense strand (Metabion, Munich, Germany). Screening was initially carried out at one concentration (10 μM) in a 20-μl reaction buffer. A dose-response assay was subsequently carried out at six concentrations (1.25 nM, 10 nM, 100 nM, 1 μM, 10 μM, and 50 μM) to determine the IC_50_ and DSS values. All reaction mixtures contained 50 nM CSR3 and 375 nM labeled siRNA in 384-well black flat-bottom microplates (Corning, NY, USA) and were prepared using a BioTek MultiFlo FX dispenser with single-channel random access dispenser cassettes (BioTek, Winooski, VT, USA). The plates were read with a PHERAstar FS reader (BMG Labtech, Ortenberg, Germany).

### Binding affinity assay using MST.

Proteins were labeled using Red-Tris-NTA dye (NanoTemper, Munich, Germany) and resuspended in 50 μl of phosphate-buffered saline (PBS) buffer (137 mM NaCl, 2.7 mM KCl, 10 mM Na_2_HPO_4_, 2 mM KH_2_PO_4_ [pH 7.4]) with 0.05% Tween 20 to obtain a 5 μM dye solution. The labeled-protein solution containing 500 nM proteins and 40 nM dye was prepared in PBS buffer with 2% dimethyl sulfoxide (DMSO) for the assay. The 12 concentrations for each compound were obtained by 2-fold serial dilutions (0.2 μM to 400 μM). A peptide control was included to discriminate binding-specific fluorescence quenching from the loss of fluorescence due to protein precipitation. Two independent experiments were carried out in premium coated capillaries (NanoTemper, Munich, Germany) using a Monolith NT.Automated instrument (NanoTemper) with the power set at high (80%), the light-emitting diode (LED) power (pico red) set at 5%, and the on-time set at 20 s. The dissociation constant (*K_d_*) was determined using the MO.Affinity Analysis program (NanoTemper).

### Binding affinity assay using SPR.

CSR3 was purified as described above and stored in PBS buffer. SPR was performed on a BiacoreT100 instrument (GE Healthcare) using a sensor S CM5 chip (GE Healthcare). CSR3 (10 ng/μl) was immobilized using the standard amine-coupling method according to the manufacturer’s instructions with immobilization buffer (10 mM sodium acetate [pH 4]); the final response unit (RU) value was 11,459. Compounds at a single concentration (100 μM) or consisting of 2-fold serial dilutions (3 μM to 200 μM) were flowed over the sensor surface using PBS buffer with 0.01 M HEPES, 0.05% surfactant P20, and 2% DMSO. Compounds were tested from lower to higher concentrations at 25°C, and injection and dissociation were performed at a flow rate of 30 μl/min for 60 s and 300 s, respectively. To eliminate bulk interference by DMSO, a solvent correction consisting of DMSO at concentrations ranging from 1.5% to 2.8% was carried out for every 30 samples. Steady-state affinity (*K_D_*) values were determined using BiacoreT100 Evaluation software, v.2.04 (GE Healthcare).

### Plant material, growth conditions, and phenotyping.

Sweetpotato plants cultivar 'Huachano' (accession CIP420065) were side-graft inoculated with both SPFMV (East African strain isolate Nam1) and SPCSV-Ug (East African serotype 2) as described previously (62). Plantlets were propagated by taking single-node stems grown in a plant culture medium (33). Next, plantlets with newly formed roots were transferred to glass tubes (18 by 150 mm) containing 10 ml of medium supplemented with either 50 μM compound (diluted in DMSO; final DMSO concentration, 0.1%) or only 0.1% DMSO as a control. For plant experiments in soil, plantlets were transferred to pots (6 by 6 by 10 cm) filled with a mixture of sand, humus, and washed soil. After 1 week, plants were treated by foliar spraying using either individual compounds at a single concentration (10 μM) (treatment) or water (control/mock) twice a week for 1 month. All plants were grown at 22°C with 60% humidity and a 16-h light/8-h dark photoperiod for 28 days in culture medium and for 41 days in soil. The Finnish National Plant Phenotyping Infrastructure (NaPPI) was used to monitor plant viral disease symptoms as described in our previous study (33). Four compounds were tested with the phenotyping platform in two independent batches, each of which included five biological replicates.

### Virus accumulation assay with RT-qPCR.

Leaf samples were collected from plants grown in culture medium or soil for 28 or 41 days, respectively, and frozen in liquid nitrogen. Total RNA was extracted using the Spectrum plant total RNA kit (Sigma-Aldrich). First-strand cDNA was synthesized using the Transcriptor first-strand cDNA synthesis kit (Roche, Basel, Switzerland). Gene expression was measured in a final volume of 10 μl (containing 2 μl 10-fold-diluted cDNA, 5 μl SYBR green I master mix [Roche], and 2.5 μM primers) using the LightCycler 480 instrument II (Roche). All RT-qPCR experiments were conducted in triplicate on three and five biological replicates from plants grown in culture medium and soil, respectively. The primer list can be found in our previous study (33).

### Data analysis.

For HTS, the activity of CSR3 was calculated by measuring the change in fluorescence as a function of the reaction time using MARS Data Analysis software (BMG Labtech). To evaluate the CSR3 inhibition efficiency of individual compounds, the PI of each compound was calculated using the slope values for the sample, positive control, and negative control according to the following equation: PI = 100 × [1 − (sample − positive)/(negative − positive)]%. IC_50_ and DSS values were calculated using concentration-specific PI values according to a previous study (31). Relative gene expression was calculated using the classical 2^−ΔΔ^*^CT^* method (63).

## References

[1] Clark CA, Davis JA, Abad JA, Cuellar WJ, Fuentes S, Kreuze JF, Gibson RW, Mukasa SB, Tugume AK, Tairo FD, Valkonen JP. 2012. Sweetpotato viruses: 15 years of progress on understanding and managing complex diseases. Plant Dis 96:168–185. 10.1094/PDIS-07-11-0550. 30731810

[2] Gibson RW, Kreuze JF. 2015. Degeneration in sweetpotato due to viruses, virus-cleaned planting material and reversion: a review. Plant Pathol 64:1–15. 10.1111/ppa.12273.

[3] Njeru RW, Mburu MWK, Cheramgoi E, Gibson RW, Kiburi ZM, Obudho E, Yobera D. 2004. Studies on the physiological effects of viruses on sweet potato yield in Kenya. Ann Appl Biol 145:71–76. 10.1111/j.1744-7348.2004.tb00360.x.

[4] Untiveros M, Fuentes S, Salazar LF. 2007. Synergistic interaction of Sweet potato chlorotic stunt virus (Crinivirus) with carla-, cucumo-, ipomo-, and potyviruses infecting sweet potato. Plant Dis 91:669–676. 10.1094/PDIS-91-6-0669. 30780474

[5] Ngailo S, Shimelis H, Sibiya J, Mtunda K, Mashilo J. 2019. Genotype-by-environment interaction of newly-developed sweet potato genotypes for storage root yield, yield-related traits and resistance to sweet potato virus disease. Heliyon 5:e01448. 10.1016/j.heliyon.2019.e01448. 30976707 PMC6441836

[6] Okada Y, Kobayashi A, Tabuchi H, Kuranouchi T. 2017. Review of major sweetpotato pests in Japan, with information on resistance breeding programs. Breed Sci 67:73–82. 10.1270/jsbbs.16145. 28465670 PMC5407922

[7] Wang Q, Valkonen JP. 2009. Cryotherapy of shoot tips: novel pathogen eradication method. Trends Plant Sci 14:119–122. 10.1016/j.tplants.2008.11.010. 19217342

[8] Qu F. 2010. Plant viruses versus RNAi: simple pathogens reveal complex insights on plant antimicrobial defense. Wiley Interdiscip Rev RNA 1:22–33. 10.1002/wrna.7. 21956904

[9] Agrawal N, Dasaradhi P, Mohmmed A, Malhotra P, Bhatnagar RK, Mukherjee SK. 2003. RNA interference: biology, mechanism, and applications. Microbiol Mol Biol Rev 67:657–685. 10.1128/mmbr.67.4.657-685.2003. 14665679 PMC309050

[10] Li F, Wang A. 2019. RNA-targeted antiviral immunity: more than just RNA silencing. Trends Microbiol 27:792–805. 10.1016/j.tim.2019.05.007. 31213342

[11] Burgyan J, Havelda Z. 2011. Viral suppressors of RNA silencing. Trends Plant Sci 16:265–272. 10.1016/j.tplants.2011.02.010. 21439890

[12] Niehl A, Wyrsch I, Boller T, Heinlein M. 2016. Double-stranded RNAs induce a pattern-triggered immune signaling pathway in plants. New Phytol 211:1008–1019. 10.1111/nph.13944. 27030513

[13] Cuellar WJ, Kreuze JF, Rajamaki ML, Cruzado KR, Untiveros M, Valkonen JP. 2009. Elimination of antiviral defense by viral RNase III. Proc Natl Acad Sci U S A 106:10354–10358. 10.1073/pnas.0806042106. 19515815 PMC2694682

[14] Tugume AK, Amayo R, Weinheimer I, Mukasa SB, Rubaihayo PR, Valkonen JP. 2013. Genetic variability and evolutionary implications of RNA silencing suppressor genes in RNA1 of sweet potato chlorotic stunt virus isolates infecting sweetpotato and related wild species. PLoS One 8:e81479. 10.1371/journal.pone.0081479. 24278443 PMC3838340

[15] Weinheimer I, Jiu Y, Rajamaki ML, Matilainen O, Kallijarvi J, Cuellar WJ, Lu R, Saarma M, Holmberg CI, Jantti J, Valkonen JP. 2015. Suppression of RNAi by dsRNA-degrading RNaseIII enzymes of viruses in animals and plants. PLoS Pathog 11:e1004711. 10.1371/journal.ppat.1004711. 25747942 PMC4352025

[16] Kreuze JF, Savenkov EI, Valkonen JPT. 2002. Complete genome sequence and analyses of the subgenomic RNAs of Sweet potato chlorotic stunt virus reveal several new features for the genus Crinivirus. J Virol 76:9260–9270. 10.1128/jvi.76.18.9260-9270.2002. 12186910 PMC136465

[17] Du Z, Lee JK, Tjhen R, Stroud RM, James TL. 2008. Structural and biochemical insights into the dicing mechanism of mouse Dicer: a conserved lysine is critical for dsRNA cleavage. Proc Natl Acad Sci U S A 105:2391–2396. 10.1073/pnas.0711506105. 18268334 PMC2268147

[18] Kreuze JF, Savenkov EI, Cuellar W, Li X, Valkonen JP. 2005. Viral class 1 RNase III involved in suppression of RNA silencing. J Virol 79:7227–7238. 10.1128/JVI.79.11.7227-7238.2005. 15890961 PMC1112141

[19] Kharrat A, Macias M, Gibson T, Nilges M, Pastore A. 1995. Structure of the dsRNA binding domain of E. coli RNase III. EMBO J 14:3572–3584. 10.1002/j.1460-2075.1995.tb07363.x. 7628457 PMC394425

[20] Court DL, Gan J, Liang YH, Shaw GX, Tropea JE, Costantino N, Waugh DS, Ji X. 2013. RNase III: genetics and function; structure and mechanism. Annu Rev Genet 47:405–431. 10.1146/annurev-genet-110711-155618. 24274754 PMC6311387

[21] Roy A, Kucukural A, Zhang Y. 2010. I-TASSER: a unified platform for automated protein structure and function prediction. Nat Protoc 5:725–738. 10.1038/nprot.2010.5. 20360767 PMC2849174

[22] Nicholson AW. 2014. Ribonuclease III mechanisms of double-stranded RNA cleavage. Wiley Interdiscip Rev RNA 5:31–48. 10.1002/wrna.1195. 24124076 PMC3867540

[23] Akey DL, Berger JM. 2005. Structure of the nuclease domain of ribonuclease III from M. tuberculosis at 2.1 Å. Protein Sci 14:2744–2750. 10.1110/ps.051665905. 16155207 PMC2253305

[24] Wang L, Saarela J, Poque S, Valkonen JPT. 2020. Development of FRET-based high-throughput screening for viral RNase III inhibitors. Mol Plant Pathol 21:961–974. 10.1111/mpp.12942. 32436305 PMC7280029

[25] Good AC, Oprea TI. 2008. Optimization of CAMD techniques 3. Virtual screening enrichment studies: a help or hindrance in tool selection? J Comput Aided Mol Des 22:169–178. 10.1007/s10822-007-9167-2. 18188508

[26] Friesner RA, Murphy RB, Repasky MP, Frye LL, Greenwood JR, Halgren TA, Sanschagrin PC, Mainz DT. 2006. Extra precision glide: docking and scoring incorporating a model of hydrophobic enclosure for protein-ligand complexes. J Med Chem 49:6177–6196. 10.1021/jm051256o. 17034125

[27] Weinheimer I, Boonrod K, Moser M, Wassenegger M, Krczal G, Butcher SJ, Valkonen JP. 2014. Binding and processing of small dsRNA molecules by the class 1 RNase III protein encoded by sweet potato chlorotic stunt virus. J Gen Virol 95:486–495. 10.1099/vir.0.058693-0. 24187016

[28] Liang YH, Lavoie M, Comeau MA, Abou Elela S, Ji X. 2014. Structure of a eukaryotic RNase III postcleavage complex reveals a double-ruler mechanism for substrate selection. Mol Cell 54:431–444. 10.1016/j.molcel.2014.03.006. 24703949 PMC4019767

[29] Gan J, Tropea JE, Austin BP, Court DL, Waugh DS, Ji X. 2006. Structural insight into the mechanism of double-stranded RNA processing by ribonuclease III. Cell 124:355–366. 10.1016/j.cell.2005.11.034. 16439209

[30] Zhang JH, Chung TD, Oldenburg KR. 1999. A simple statistical parameter for use in evaluation and validation of high throughput screening assays. J Biomol Screen 4:67–73. 10.1177/108705719900400206. 10838414

[31] Yadav B, Pemovska T, Szwajda A, Kulesskiy E, Kontro M, Karjalainen R, Majumder MM, Malani D, Murumagi A, Knowles J, Porkka K, Heckman C, Kallioniemi O, Wennerberg K, Aittokallio T. 2014. Quantitative scoring of differential drug sensitivity for individually optimized anticancer therapies. Sci Rep 4:5193. 10.1038/srep05193. 24898935 PMC4046135

[32] Kokkinos CD, Clark C. 2006. Real-time PCR assays for detection and quantification of sweetpotato viruses. Plant Dis 90:783–788. 10.1094/PD-90-0783. 30781240

[33] Wang L, Poque S, Valkonen JPT. 2019. Phenotyping viral infection in sweetpotato using a high-throughput chlorophyll fluorescence and thermal imaging platform. Plant Methods 15:116. 10.1186/s13007-019-0501-1. 31649744 PMC6805361

[34] Jones RA. 2006. Control of plant virus diseases. Adv Virus Res 67:205–244. 10.1016/S0065-3527(06)67006-1. 17027681

[35] Yu Y, Wang X, Sun H, Liang Q, Wang W, Zhang C, Bian X, Cao Q, Li Q, Xie Y, Ma D, Li Z, Sun J. 2020. Improving CRISPR‐Cas‐mediated RNA targeting and gene editing using SPLCV replicon‐based expression vectors in Nicotiana benthamiana. Plant Biotechnol J 18:1993–1995. 10.1111/pbi.13384. PMC7539982 32289196

[36] Ding SW, Voinnet O. 2007. Antiviral immunity directed by small RNAs. Cell 130:413–426. 10.1016/j.cell.2007.07.039. 17693253 PMC2703654

[37] Roth BM, Pruss GJ, Vance VB. 2004. Plant viral suppressors of RNA silencing. Virus Res 102:97–108. 10.1016/j.virusres.2004.01.020. 15068885

[38] Muhammad T, Zhang F, Zhang Y, Liang Y. 2019. RNA interference: a natural immune system of plants to counteract biotic stressors. Cells 8:38. 10.3390/cells8010038. PMC6356646 30634662

[39] Li FF, Wang AM. 2018. RNA decay is an antiviral defense in plants that is counteracted by viral RNA silencing suppressors. PLoS Pathog 14:e1007228. 10.1371/journal.ppat.1007228. 30075014 PMC6101400

[40] Hu F, Lei R, Deng YF, Wang J, Li GF, Wang CN, Li ZH, Zhu SF. 2018. Discovery of novel inhibitors of RNA silencing suppressor P19 based on virtual screening. RSC Adv 8:10532–10540. 10.1039/C8RA01311J. PMC9078884 35540466

[41] Shimura H, Fukagawa T, Meguro A, Yamada H, Oh-Hira M, Sano S, Masuta C. 2008. A strategy for screening an inhibitor of viral silencing suppressors, which attenuates symptom development of plant viruses. FEBS Lett 582:4047–4052. 10.1016/j.febslet.2008.10.046. 18996375

[42] Sagan SM, Koukiekolo R, Rodgers E, Goto NK, Pezacki JP. 2007. Inhibition of siRNA binding to a p19 viral suppressor of RNA silencing by cysteine alkylation. Angew Chem Int Ed Engl 46:2005–2009. 10.1002/anie.200603284. 17286327

[43] Mat Jalaluddin NS, Othman RY, Harikrishna JA. 2019. Global trends in research and commercialization of exogenous and endogenous RNAi technologies for crops. Crit Rev Biotechnol 39:67–78. 10.1080/07388551.2018.1496064. 30198341

[44] Dalakouras A, Wassenegger M, Dadami E, Ganopoulos I, Pappas ML, Papadopoulou K. 2020. Genetically modified organism-free RNA interference: exogenous application of RNA molecules in plants. Plant Physiol 182:38–50. 10.1104/pp.19.00570. 31285292 PMC6945881

[45] Worrall EA, Bravo-Cazar A, Nilon AT, Fletcher SJ, Robinson KE, Carr JP, Mitter N. 2019. Exogenous application of RNAi-inducing double-stranded RNA inhibits aphid-mediated transmission of a plant virus. Front Plant Sci 10:265. 10.3389/fpls.2019.00265. 30930914 PMC6429036

[46] Mitter N, Worrall EA, Robinson KE, Xu ZP, Carroll BJ. 2017. Induction of virus resistance by exogenous application of double-stranded RNA. Curr Opin Virol 26:49–55. 10.1016/j.coviro.2017.07.009. 28778033

[47] Tenllado F, Martinez-Garcia B, Vargas M, Diaz-Ruiz JR. 2003. Crude extracts of bacterially expressed dsRNA can be used to protect plants against virus infections. BMC Biotechnol 3:3. 10.1186/1472-6750-3-3. 12659646 PMC153545

[48] Konakalla NC, Kaldis A, Berbati M, Masarapu H, Voloudakis AE. 2016. Exogenous application of double-stranded RNA molecules from TMV p126 and CP genes confers resistance against TMV in tobacco. Planta 244:961–969. 10.1007/s00425-016-2567-6. 27456838

[49] Thakur CS, Jha BK, Dong B, Das Gupta J, Silverman KM, Mao H, Sawai H, Nakamura AO, Banerjee AK, Gudkov A, Silverman RH. 2007. Small-molecule activators of RNase L with broad-spectrum antiviral activity. Proc Natl Acad Sci U S A 104:9585–9590. 10.1073/pnas.0700590104. 17535916 PMC1877983

[50] Klumpp K, Hang JQ, Rajendran S, Yang Y, Derosier A, In PWK, Overton H, Parkes KEB, Cammack N, Martin JA. 2003. Two‐metal ion mechanism of RNA cleavage by HIV RNase H and mechanism‐based design of selective HIV RNase H inhibitors. Nucleic Acids Res 31:6852–6859. 10.1093/nar/gkg881. 14627818 PMC290251

[51] Friesner RA, Banks JL, Murphy RB, Halgren TA, Klicic JJ, Mainz DT, Repasky MP, Knoll EH, Shelley M, Perry JK, Shaw DE, Francis P, Shenkin PS. 2004. Glide: a new approach for rapid, accurate docking and scoring. 1. Method and assessment of docking accuracy. J Med Chem 47:1739–1749. 10.1021/jm0306430. 15027865

[52] Miyashita S. 2018. Studies on replication and evolution mechanisms of plant RNA viruses. J Gen Plant Pathol 84:427–428. 10.1007/s10327-018-0806-3.

[53] Cai X, Zheng W, Li Z. 2019. High-throughput screening strategies for the development of anti-virulence inhibitors against Staphylococcus aureus. Curr Med Chem 26:2297–2312. 10.2174/0929867324666171121102829. 29165063

[54] Klostermeier D, Sears P, Wong CH, Millar DP, Williamson JR. 2004. A three-fluorophore FRET assay for high-throughput screening of small-molecule inhibitors of ribosome assembly. Nucleic Acids Res 32:2707–2715. 10.1093/nar/gkh588. 15148358 PMC419595

[55] Entzian C, Schubert T. 2016. Studying small molecule-aptamer interactions using microscale thermophoresis (MST). Methods 97:27–34. 10.1016/j.ymeth.2015.08.023. 26334574

[56] Patching SG. 2014. Surface plasmon resonance spectroscopy for characterisation of membrane protein-ligand interactions and its potential for drug discovery. Biochim Biophys Acta 1838:43–55. 10.1016/j.bbamem.2013.04.028. 23665295

[57] Retra K, Irth H, van Muijlwijk-Koezen JE. 2010. Surface plasmon resonance biosensor analysis as a useful tool in FBDD. Drug Discov Today Technol 7:e181–e187. 10.1016/j.ddtec.2010.11.012.

[58] Selberg S, Blokhina D, Aatonen M, Koivisto P, Siltanen A, Mervaala E, Kankuri E, Karelson M. 2019. Discovery of small molecules that activate RNA methylation through cooperative binding to the METTL3-14-WTAP complex active site. Cell Rep 26:3762–3771.e5. 10.1016/j.celrep.2019.02.100. 30917327

[59] Suree N, Yi SW, Thieu W, Marohn M, Damoiseaux R, Chan A, Jung ME, Clubb RT. 2009. Discovery and structure-activity relationship analysis of Staphylococcus aureus sortase A inhibitors. Bioorg Med Chem 17:7174–7185. 10.1016/j.bmc.2009.08.067. 19781950 PMC2888031

[60] Katoh K, Misawa K, Kuma K, Miyata T. 2002. MAFFT: a novel method for rapid multiple sequence alignment based on fast Fourier transform. Nucleic Acids Res 30:3059–3066. 10.1093/nar/gkf436. 12136088 PMC135756

[61] Yang J, Yan R, Roy A, Xu D, Poisson J, Zhang Y. 2015. The I-TASSER suite: protein structure and function prediction. Nat Methods 12:7–8. 10.1038/nmeth.3213. 25549265 PMC4428668

[62] Wang QC, Valkonen JP. 2008. Elimination of two viruses which interact synergistically from sweetpotato by shoot tip culture and cryotherapy. J Virol Methods 154:135–145. 10.1016/j.jviromet.2008.08.006. 18786569

[63] Rao X, Huang X, Zhou Z, Lin X. 2013. An improvement of the 2(−delta delta CT) method for quantitative real-time polymerase chain reaction data analysis. Biostat Bioinforma Biomath 3:71–85. 25558171 PMC4280562

