# Supplementary material for: In Vitro Identification and In Vivo Confirmation of Inhibitors for Sweet Potato Chlorotic Stunt Virus RNA Silencing Suppressor, a Viral RNase III
Source: J Virol. 2021 May 24;95(12):e00107-21. doi: 10.1128/JVI.00107-21 (PMC8315922; doi:10.1128/JVI.00107-21)
Supplement: Supplementary file 2 [file jvi.00107-21-s0002.pdf]

Table S1 Selected 6,400 compounds and their docking scores using virtual screening

Table S2 PI values of the 6,622 compounds

Table S3 Dose-response results of 112 compounds from three repeats (FIMM 1, FIMM 2, and Comm 1)

Table S4 MST binding affinity results of the 36 compounds

Table S5 SPR binding affinity results of 36 compounds

Table S6 Compounds' effects on both SPFMV and SPCSV accumulation in plants grown in culture medium.

Table S7 Information of the selected five inhibitors, including their molecular formulas, suppliers, molecular weights, SMILES.
